# Supplementary material for: The interactome of intact mitochondria by cross-linking mass spectrometry provides evidence for coexisting respiratory supercomplexes
Source: Mol Cell Proteomics. 2017 Dec 8;17(2):216–32. doi: 10.1074/mcp.RA117.000470 (PMC5795388; doi:10.1074/mcp.RA117.000470)
Supplement: Supplemental Data [file supp_RA117.000470_133922_0_supp_23978_fzffwf.zip › spectra_annotation/mito_spectra_annotation/214-2-6-1-6-1.pdf]

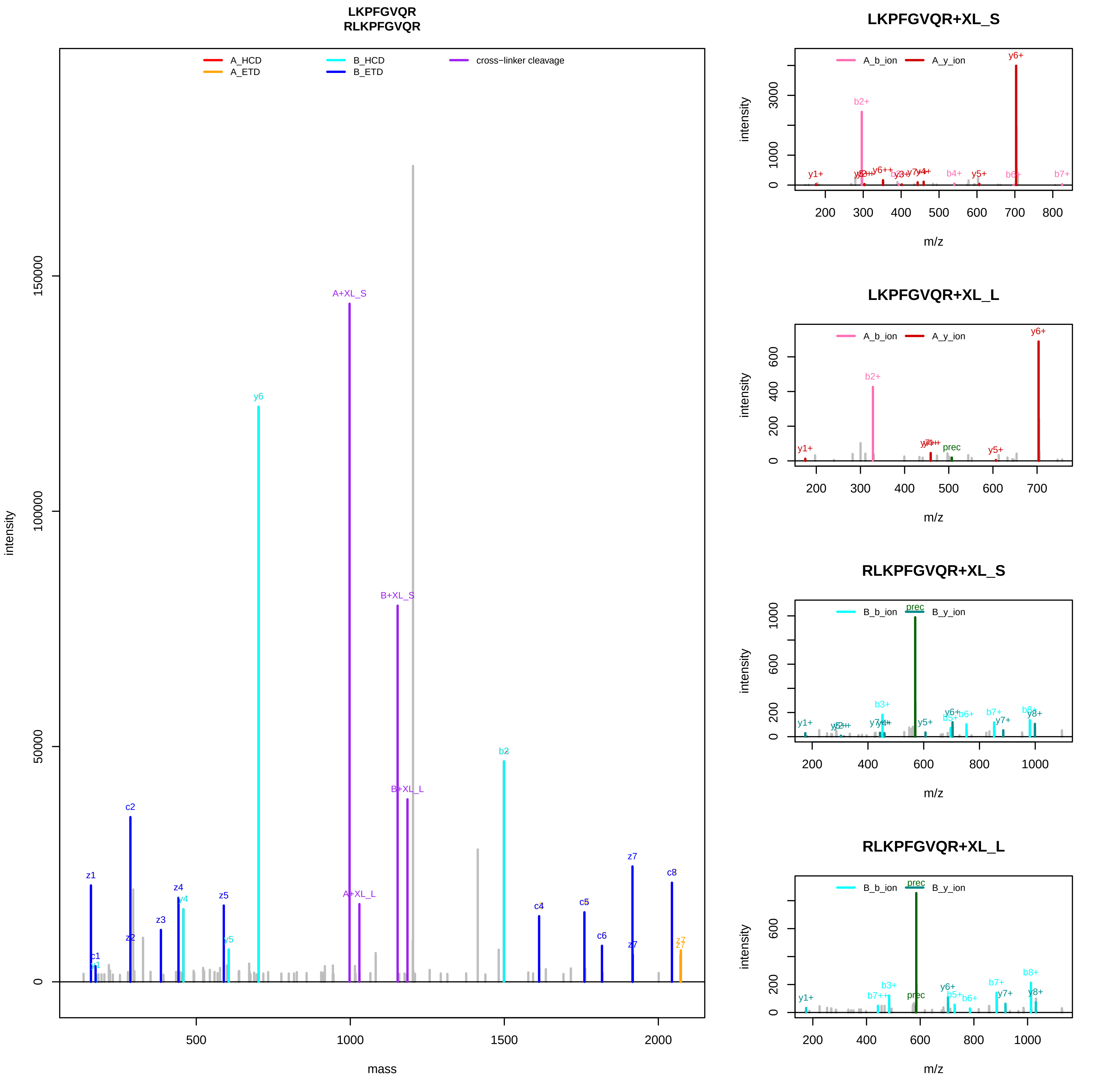
LKPFGVQR
RLKPFGVQR
A_HCD
A_ETD
B_HCD
B_ETD
cross−linker cleavage
A+XL_S
B+XL_S
B+XL_L
A+XL_L
y6
b2
c2
z1
c1
y1
z2
z3
z4
y4
z5
y5
c4
c5
c6
z7
c8
intensity
mass
0
50000
100000
150000
500
1000
1500
2000
LKPFGVQR+XL_S
A_b_ion
A_y_ion
y6+
b2+
y1+
y6++
b4+
y5+
b6+
b7+
m/z
LKPFGVQR+XL_L
prec
RLKPFGVQR+XL_S
B_b_ion
B_y_ion
b3+
y6+
b5+
b6+
b7+
y7+
b8+
y8+
RLKPFGVQR+XL_L
b7++
y1+
